# Dopaminergic and Clinical Correlates of Pathological Gambling in Parkinson’s Disease: A Case Report

**DOI:** 10.3389/fnbeh.2013.00095

**Published:** 2013-07-29

**Authors:** Mette Buhl Callesen, K. V. Hansen, A. Gjedde, J. Linnet, A. Møller

**Affiliations:** ^1^Department of Nuclear Medicine and PET-Centre, Aarhus University Hospital, Aarhus, Denmark; ^2^Centre of Functionally Integrative Neuroscience, Aarhus University, Aarhus, Denmark; ^3^Department of Neuroscience and Pharmacology, University of Copenhagen, Copenhagen, Denmark; ^4^Research Clinic on Gambling Disorders, Aarhus University Hospital, Aarhus, Denmark

**Keywords:** Parkinson’s disease, pathological gambling, impulse control disorders, decision-making, dopamine

## Abstract

Dopaminergic medication for motor symptoms in Parkinson’s disease (PD) recently has been linked with impulse control disorders, including pathological gambling (PG), which affects up to 8% of patients. PG often is considered a behavioral addiction associated with disinhibition, risky decision-making, and altered striatal dopaminergic neurotransmission. Using [^11^C]raclopride with positron emission tomography, we assessed dopaminergic neurotransmission during Iowa Gambling Task performance. Here we present data from a single patient with PD and concomitant PG. We noted a marked decrease in [^11^C]raclopride binding in the left ventral striatum upon gambling, indicating a gambling-induced dopamine release. The results imply that PG in PD is associated with a high dose of dopaminergic medication, pronounced motor symptomatology, young age at disease onset, high propensity for sensation seeking, and risky decision-making. Overall, the findings are consistent with the hypothesis of medication-related PG in PD and underscore the importance of taking clinical variables, such as age and personality, into account when patients with PD are medicated, to reduce the risk of PG.

## Introduction

Parkinson’s disease (PD) is a neurodegenerative disorder associated with a progressive nigrostriatal and mesocorticolimbic dopamine depletion, resulting in core motor symptoms of resting tremor, bradykinesia, rigidity, and postural instability. Recent evidence established that dopaminergic medication administered to relieve motor symptoms in PD may have detrimental effects on cognitive functioning, including executive functions, decision-making, and impulse control (Cools, 2006; Frank et al., 2007; Rowe et al., 2008; Cools et al., 2009; Poletti et al., 2010). In early PD, dopamine depletion is restricted to the dorsal striatum, leaving the ventral striatum relatively intact. Thus, medication doses necessary to remedy the dopaminergic loss in the dorsal striatum may excessively stimulate the ventral striatum, potentially leading to unusual repetitive and compulsive behaviors and impulse control disorders, at least in a subgroup of PD patients (Cools, 2006; Rowe et al., 2008; Cools et al., 2009; Poletti et al., 2010; Weintraub and Nirenberg, 2012).

Pathological gambling (PG) is an impulse control disorder characterized by recurrent maladaptive gambling behavior despite personal, social, and financial consequences (APA, 1994), affecting up to 8% of PD patients treated with dopamine agonists (Weintraub et al., 2010), a notably higher prevalence compared to a prevalence of PG of 0.4–1.6% in the general population (Schreiber et al., 2011). This particular complication to the treatment most often occur subsequent to treatment initiation, or dosage increase, and tend to improve or fully alleviate following reduction or discontinuation of the dopamine agonists (Callesen et al., 2013). Besides dopamine agonists, additional risk factors include young age at PD onset (often in early forties), male gender, personal or family history of addictive behaviors, genetic factors, depressive symptoms, and an impulsive and novelty seeking personality (Voon et al., 2007, 2011a; Wu et al., 2009; Claassen et al., 2011; Joutsa et al., 2012; Lee et al., 2012; Poletti and Bonuccelli, 2012; Weintraub and Nirenberg, 2012; Callesen et al., 2013; Kim et al., 2013). Often, PG is held to be a behavioral addiction associated with temporal discounting, disinhibition, and risky decision-making, to which the Iowa Gambling Task (IGT) (Bechara et al., 1994; Grant et al., 2000; Petry, 2001; Manes et al., 2002; Goudriaan et al., 2005, 2006; Linnet et al., 2006, 2010, 2011a; Peterson et al., 2010; van Holst et al., 2010) is applicable.

Commonly used as an experimental decision-making task, the IGT mimics complex real-life decision-making processes embedding factors of ambiguity, anticipation, reward, and punishment (Bechara et al., 1994). Performance on the IGT is a sensitive measure of impaired decision-making in diverse neurological and psychiatric conditions (Bechara et al., 1994). Patients with frontal lesions, patients suffering from substance use disorders, and pathological gamblers have demonstrated a preference for short-term gains in spite of larger long-term losses when they perform the IGT (Bechara et al., 1994; Grant et al., 2000; Petry, 2001; Manes et al., 2002; Goudriaan et al., 2005, 2006; Linnet et al., 2006, 2010, 2011a; Peterson et al., 2010; van Holst et al., 2010). Furthermore, Linnet et al. (2010, 2011a) recently showed that impaired IGT performance in pathological gamblers is associated with an increased dopamine release in the ventral striatum, which is crucial in reinforcement of behavior – including disadvantageous behavior (Linnet et al., 2011b, 2012).

Likewise, studies of decision-making in PD with the IGT reported poorer IGT performance in PD patients than in healthy controls (Perretta et al., 2005; Mimura et al., 2006; Pagonabarraga et al., 2007; Kobayakawa et al., 2008; Ibarretxe-Bilbao et al., 2009; Rossi et al., 2010; Poletti et al., 2011; Gescheidt et al., 2012), also early in the disease (Perretta et al., 2005; Ibarretxe-Bilbao et al., 2009). In contrast, Euteneuer et al. (2009) showed intact IGT performance in PD patients, and Gescheidt et al. (2012) demonstrated only slightly reduced IGT performance in patients with early-onset PD compared to healthy age-matched controls. However, compared to controls, patients did not develop an effective decision-making strategy and tended to change their deck preferences more frequently despite intact executive functions (Gescheidt et al., 2012). In addition, Poletti et al. (2010) recently showed similar IGT performance in *de novo* PD patients and healthy age-matched controls, suggesting that decision-making deficits in early PD emerge after dopaminergic medication rather than as a result of PD *per se*.

In few studies, did the authors compare IGT performance of PD patients with and without PG (Rossi et al., 2010; Bentivoglio et al., 2012). Rossi et al. (2010) found that PD patients with PG performed significantly worse on the IGT than PD patients without PG, whereas Bentivoglio et al. (2012) noted only a trend toward poorer performance characterized by more risky choices and greater monetary loss in patients with impulse control disorders compared to PD controls.

The decision-making impairments observed in PD patients with and without PG are held widely to be related to deficits in the fronto-striatal circuitry (Brand et al., 2004; Perretta et al., 2005; Cools, 2006; Mimura et al., 2006; Pagonabarraga et al., 2007; Kobayakawa et al., 2008; Rowe et al., 2008; Cools et al., 2009; Euteneuer et al., 2009; Ibarretxe-Bilbao et al., 2009; Gleichgerrcht et al., 2010; Poletti et al., 2010; Rossi et al., 2010). The involvement of the ventral striatum, which appears especially important in the underlying dopaminergic dysfunctions of PG, is uncertain in medication-induced PG, particularly as related to dopamine agonists, in PD (Perretta et al., 2005; Cools, 2006; Pagonabarraga et al., 2007; Rowe et al., 2008; Cools et al., 2009; Linnet et al., 2010, 2011a; Peterson et al., 2010; Poletti et al., 2010). While the dopaminergic mechanism of the increased risk of PG in PD remains unknown, Steeves et al. (2009) find support for the involvement of the ventral striatum in PG in PD by demonstrating that PD patients with PG release significantly more dopamine in the ventral striatum during gambling compared to PD patients without PG.

This study was designed to compare dopaminergic neurotransmission during gambling in 40- to 65-year-old male PD patients with PG compared to PD patients without PG, pathological gamblers without PD, and healthy controls matched on age and gender. Using [^11^C]raclopride positron emission tomography (PET), we assessed changes in dopamine occupancy between a baseline and an active gambling condition testing the hypothesis of increased dopamine release in the ventral striatum upon gambling by PD patients with PG and by pathological gamblers. Due to difficult recruiting of subjects, particularly PD patients with PG, the study was designed to allow for a continuous data collection without methodological modifications prohibiting replication. Here we present data from the first PD patient with PG and discuss the results with reference to the results from four PD controls without PG.

## Methods and Materials

### Subjects

The participant with PG was a 56-year-old married man, GL, with a 15-year history of PD recruited through the Danish Parkinson Association. He had a history of recreational gambling, but his gambling behavior had been pathological for the past 11 years. He had no history of smoking or drug use. The four PD controls were male patients aged 41–59 years (mean = 50 years) with a history of PD of 5–7 years (mean = 6 years). They had no history of gambling, nor smoking, or drug use. All subjects gave written informed consent before entering the study, which was approved by the local ethical committee and performed in accordance with the Helsinki II declaration.

### Clinical assessment

Parkinson’s disease variables were evaluated using a demographic and clinical questionnaire consisting of 24 items including motor symptomatology (10 yes/no questions about tremor, ON/OFF periods, postural instability, bradykinesia, rigidity, dyskinesia, freezing, problems swallowing, and muscle pain or numbness) and medication. We evaluated the patients’ gambling behavior the South Oaks Gambling Screen (SOGS) (Lesieur and Blume, 1987) and the Structured Clinical Interview for DSM-IV Axis I disorders (SCID-I) (First et al., 2002), which also screened for additional Axis I psychopathology including mood and anxiety disorders and substance use disorders. The SOGS is a self-administered questionnaire ranging from 0 to 20, assessing presence and severity of gambling symptoms. A score of 5 or more indicates probable PG (Lesieur and Blume, 1987). The SOGS has shown good reliability and validity with the DSM-IV criteria for PG (Stinchfield, 2002). Furthermore, we screened for depressive symptomatology using the 15-item Geriatric Depression Scale (GDS) (Sheikh and Yesavage, 1986; Burke et al., 1991; Djernes et al., 2004). The GDS is a 15-item forced-choice questionnaire estimating severity in symptoms of depression in elderly that has been validated for both younger and older patients with PD (Weintraub et al., 2006a, 2007). A score of 5 or above indicates probable depression with a higher score indicating increased severity in symptoms. Finally, the subjects answered the Zuckerman Sensation Seeking Scale (Zuckerman, 1994), which is a 40-item forced-choice questionnaire assessing the personality trait sensation seeking characterized by disinhibition, thrill and adventure seeking, experience seeking, and boredom susceptibility (Zuckerman, 1994). Within the general population, the Sensation Seeking Score is normally distributed around 20 with a standard deviation around 5 (Zuckerman, 1994).

### IGT paradigm

The IGT is a card game consisting of four decks of cards labeled A, B, C, and D in the first round, K, L, M, and N in the second round, and Q, R, S, and T in the third round. In each round, two decks are advantageous decks leading to an overall gain, whereas two decks are disadvantageous leading to an overall loss. For the purpose of the current study, we focused on performance in the ABCD round, in which decks A and B are disadvantageous decks characterized by large immediate rewards and at unpredictable time points even higher punishments leading to a net loss. In contrast, decks C and D are advantageous decks associated with smaller immediate rewards and even smaller delayed losses resulting in a net gain over time. Besides the monetary outcome the IGT provides a measure of performance, an IGT score, which is calculated by subtracting the number of disadvantageous selections from advantageous selections [(C + D)–(A + B)] (Denburg et al., 2006). Here, we used a computerized version of the IGT optimized for use with PET, which was presented for the subjects in the scanner via an overhead monitor. The participants made 100 selections in each round using a right-handed computer mouse, and each selection was followed by varying monetary gains and/or losses as described above.

### Experimental procedure

The subjects underwent two PET scans with a Siemens HRRT PET scanner in 3D acquisition mode during both of which dynamic emission recordings were obtained in 23 frames of increasing duration for 60 min following an i.v. bolus injection of the radioligand [^11^C]raclopride (mean: 312.6 MBq, range: 225–340 MBq). For each condition, a brief attenuation scan was obtained just prior to the dynamic scan. In the first 60-min baseline condition the computer automatically informed the subject which card to pick from the decks. In the second 60-min active gambling condition the participant had to make his own decisions throughout the 100 selections. Following PET scanning, we obtained T1-weighted anatomical 1.2 Miami 3D magnetic resonance (MR) images in a 3.0 T GE MR scanner to enable co-registration with PET images.

### Image analysis

Emission recordings summed over the whole hour of PET scanning for both conditions were individually registered to the native MR images using the Montreal Neurological Institute (MNI) toolbox (Collins et al., 1994). Subsequently, the MR/PET correlated images were transformed into a common stereotaxic coordinate space (Talairach and Tournoux, 1988), and anatomical volumes of interest were used to extract time-activity-curves (TACs) from the dynamic PET images for each subject and each scan. Using cerebellar TACs as reference, we obtained voxel-wise maps of [^11^C]raclopride binding potentials (BP) for both the baseline (BP_baseline_) and the active gambling condition (BP_gambling_). From these maps we extracted the average BP value for each region of interest, putamen, caudate nucleus, and ventral striatum, left and right hemisphere separately, using the Simplified Reference Tissue Model (SRTM) (Lammertsma and Hume, 1996). The BP of a given radioligand, in this case [^11^C]raclopride, is an estimate of receptor availability, i.e., an index of the number of receptors available for binding (Gjedde et al., 2005).

We calculated changes in raclopride binding potentials (*Δ*BP) for each region of interest, for both sessions. The change in binding potential upon gambling normalized to the baseline binding potential was calculated as: *Δ*BP = (BP_gambling_–BP_baseline_)/BP_baseline_. Thus, a gambling-evoked decrease of [^11^C]raclopride binding indicates an increase in dopamine occupancy, associated with an increase in the extracellular dopamine concentration, a decreased affinity of the receptors toward dopamine, a decreased number of receptors, or two or all of the above combined. Conversely, an IGT-induced increase in BP reflects a decline in dopamine occupancy.

## Results

### Clinical assessment

At the time of participation, GL received 300 mg/day of levodopa controlled release, 8 mg/day of cabergoline, and 600 mg/day of entacapone, converted into a total levodopa equivalent daily dose (LEDD) of 857.6 mg/day using the standard conversion factors described by Tomlinson et al. (2010). After 3 years, we had the chance to examine GL again. At that time he no longer received levodopa and only 1 mg/day rasagiline and 4 mg/day ropinirole equal to a total LEDD of 180 mg/day. However, his gambling problem was persistent. PD controls received levodopa, pramipexole, ropinirole, rotigotin, and rasagiline equivalent to a mean total LEDD of 797.4 mg/day. The results of the clinical evaluation are summarized in Table 1.

**Table 1:** **Clinical assessment of medication, depressive symptomatology, gambling severity, and sensation seeking**.

	GL day 1	GL follow-up	PD controls mean (range)
Total LEDD (mg/day)	857.6	180	797.4 (360–1114.5)
DA LEDD (mg/day)	533.6	80	196.3 (60–315)
Motor symptoms (0–10)	6	10	4 (2–6)
SOGS	17	–	0
Current PG	Yes	Yes	No
GDS	3	3	1 (0–3)
Sensation seeking score	27	–	17 (11–23)

*Pu, putamen; Cn, caudate nucleus; Vst, ventral striatum; BP, binding potential; ΔBP, change in binding potential*.

### IGT performance

We calculated the overall IGT score and evaluated GL’s IGT performance across 100 selections. He predominantly picked cards from deck D leading to an overall positive result, but displayed a preference for deck B as well. Of 100 choices he picked 13 cards from deck A, 30 cards from deck B, 20 cards from deck C, and 37 cards from deck D, resulting in an IGT score of 14. Overall, PD controls showed a preference for deck D, though they tended to pick cards from decks B, C, and D equally often leading to a positive mean IGT score of 17.5 (range: −8 to 62). On average, PD controls picked 15 cards from deck A (range: 4–29), 27 from deck B (range: 13–48), 23 from deck C (range: 1–46), and 35 from deck D (range: 25–47).

### PET imaging

**Figure 1:**
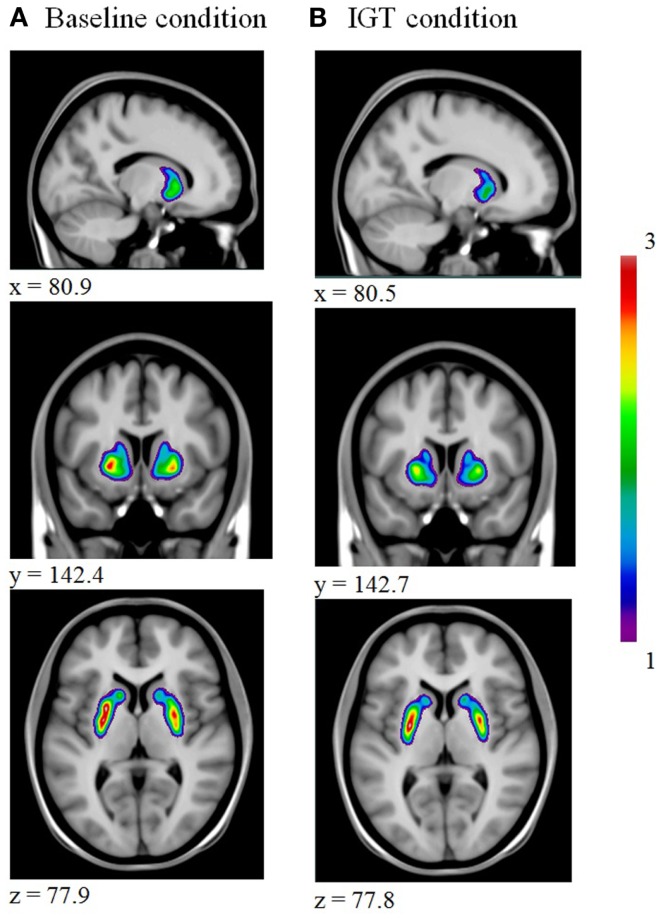
**Illustrates a decrease in striatal [^11^C]raclopride binding from baseline (A) to IGT performance (B)**.

For all subjects, we obtained parametric voxel-wise maps of [^11^C]raclopride BP for both the baseline and the IGT condition as illustrated in Figure 1.

From the parametric maps we extracted the average BP for each region of interest, putamen, caudate nucleus, and ventral striatum, left and right hemisphere, for both sessions and calculated changes in [^11^C]raclopride binding for each region of interest. The BPs are summarized in Tables 2 and 3.

**Table 2:** **[^11^C]raclopride binding potentials for the putamen, caudate nucleus, and ventral striatum, left and right hemisphere in GL**.

	BP, baseline mean (SD)	BP, gambling mean (SD)	ΔBP	ΔBP in%
Pu, left	2.50 (0.96)	2.26 (0.78)	−0.24	−9.42
Pu, right	2.40 (0.82)	2.16 (0.71)	−0.24	−9.95
Cn, left	1.37 (0.73)	1.26 (0.67)	−0.11	−8.00
Cn, right	1.30 (0.71)	1.30 (0.70)	0.00	0.00
Vst, left	1.78 (0.63)	1.36 (0.54)	−0.44	−24.92
Vst, right	1.39 (0.55)	1.28 (0.49)	−0.12	−8.32

*LEDD, levodopa equivalent daily dose; DA, dopamine agonist; SOGS, South Oaks Gambling Screen; PG, pathological gambling; GDS, geriatric depression scale; SD, standard deviation*.

**Table 3:** **Mean [^11^C]raclopride binding potentials for the putamen, caudate nucleus, and ventral striatum, left and right hemisphere in four PD patients without PG**.

	BP, baseline mean (SD)	BP, gambling mean (SD)	ΔBP mean (SD)	ΔBP in % mean (SD)
Pu, left	2.95 (1.12)	2.93 (1.39)	−0.03 (0.40)	−2.88 (0.12)
Pu, right	2.72 (0.88)	2.61 (1.19)	−0.11 (0.37)	−6.64 (0.12)
Cn, left	1.84 (0.70)	1.80 (0.88)	−0.04 (0.19)	−4.60 (0.10)
Cn, right	1.83 (0.71)	1.81 (0.94)	−0.02 (0.28)	−3.84 (0.13)
Vst, left	1.97 (0.72)	2.14 (1.02)	0.16 (0.37)	5.76 (0.15)
Vst, right	1.45 (0.36)	1.36 (0.47)	−0.08 (0.22)	−7.01 (0.17)

*Pu, putamen; Cn, caudate nucleus; Vst, ventral striatum; BP, binding potential; ΔBP, change in binding potential; SD, standard deviation*.

## Discussion

The purpose of the current case study was to investigate dopaminergic neurotransmission during IGT performance in PD patients with PG and to explore clinical correlates of PG in PD. When we first evaluated GL, his regimen included a high dose of dopaminergic medication, particularly dopamine agonists, and he displayed many symptoms of PG. Despite a change in medication and a marked reduction of the total dose, he reported persistent gambling behavior after 3 years, still spending 4 h a day on casinos, internet gambling, lotteries, scratch cards, odds, slot machines, and horse betting. Previous studies indicate that reducing the amount of dopaminergic medication, particularly dopamine agonist dose, often relieves or reduces PG symptoms (Molina et al., 2000; Dodd et al., 2005; Avanzi et al., 2006; Weintraub et al., 2006b; Weintraub and Nirenberg, 2012). In this case, gambling behavior remained, though symptoms improved following treatment changes. Unfortunately, GL displayed more motor symptoms at follow-up, probably as a consequence of disease progression but possibly also related to the dramatic reduction of medication. Overall, at baseline GL did not differ from PD controls in GDS score, but he displayed more motor symptoms, was more sensation seeking, and received a higher dose of dopamine agonists.

The primary finding of the study was the marked reduction in [^11^C]raclopride binding during gambling, relative to baseline binding, in the left ventral striatum observed in GL, suggesting a gambling-induced dopamine release. In contrast, we noted no gambling-evoked changes in BPs in PD controls, who instead displayed a small increase of [^11^C]raclopride binding in the left ventral striatum upon gambling. Similar physiological reactions to gambling have been presented in both pathological gamblers without PD (Linnet et al., 2010, 2011a; Peterson et al., 2010) and in PD patients with PG (Steeves et al., 2009) releasing significantly more dopamine in the ventral striatum during gambling than PD patients without PG. However, the findings by Steeves et al. (2009), indicated a bilateral dopamine release in the ventral striatum in PD patients with PG, but unilateral in the left ventral striatum in PD patients without PG. Unfortunately, due to the small number of subjects in this study so far, the issue of lateralization remains unresolved, yet our preliminary results support the hypothesis that this specific structure is implicated in PG and addiction. The ventral striatum is essential to reinforcement of behavior, and gambling-induced dopamine release in the ventral striatum might explain why pathological gamblers and PD patients with PG continue gambling despite personal, social, and financial consequences. In addition, compared to PD controls, GL displayed lower striatal baseline BP, which also previously was associated with an increased vulnerability to addiction and impulse control disorders (Volkow et al., 2001, 2002; Wang et al., 2001; Nader et al., 2006).

Considering GL’s decision-making strategy while performing the IGT, we noted that, despite an overall positive IGT score and a preference for deck D, he had a preference for the disadvantageous deck B. He could not completely inhibit the impulse to take the risk of a larger reward, despite the risk of an even larger punishment. A similar preference for deck B has been found in patients with early-onset PD (Gescheidt et al., 2012) who performed almost as well as healthy controls on the IGT but failed to develop an effective strategy. Instead their gambling behavior was characterized by more frequent changes in deck preferences resembling the gambling behavior we observed in both GL and PD controls. A gambling strategy characterized by cards picked almost equally often from advantageous and disadvantageous previously has been shown to elicit a larger dopamine release in the ventral striatum in pathological gamblers, relative to very poor or very good IGT performance (Linnet et al., 2012), consistent with the IGT-induced dopamine release we observed in GL. Interestingly, the dopaminergic response to gambling was absent from PD controls, despite an almost identical gambling strategy. Another possible explanation for the different ventral striatal activity between the GL and PD controls is that the dopaminergic response to gambling might be driven by the reward rate attained during task performance, which differs depending on the subjects’ decisions, rather than by the gambling aspect of the task *per se*. However, as already mentioned the groups displayed almost similar decision-making behavior, diminishing the difference in reward rates. Additionally, in contrast to the study by Steeves et al. (2009), all participants in this study experienced both rewards and penalties both at baseline and during active gambling, ensuring that only the decision-making aspect differed between conditions, thus supporting the argument of a gambling-related dopamine release. Moreover, subjects always ended up winning in the study by Steeves et al. (2009), which despite enhanced stimuli control might reduce the ecological validity of the gambling task and thus diminish the element of risky decision-making. This might explain the relatively smaller gambling-related dopamine release in PD patients with PG presented by Steeves et al. (2009) relative to a somewhat larger reduction in [^11^C]raclopride binding observed in GL. Nevertheless, in order to determine whether the different dopaminergic response between groups is determined by the actual reward rate attained, the expectation of reward, the level of excitement, or by the gambling aspect itself more participants need to be included in the study.

In addition, GL’s tendency to prefer immediate rewards and devalue delayed rewards, referred to as temporal or delayed discounting, likewise has been found in previous studies of impulse control disorders in PD (Housden et al., 2010; Voon et al., 2010, 2011a,b), linking the disinhibited behavior to dopaminergic medication, particularly the dopamine agonists (Housden et al., 2010; Voon et al., 2010, 2011b; Voon and Dalley, 2011), as the tendency toward risky decision-making may result from the high dose of dopamine agonists. While the total LEDD was not very different between GL and PD controls, GL was prescribed a very high dopamine agonist LEDD compared to PD controls. Dopamine agonists previously were associated with increased novelty seeking (Bodi et al., 2009), and hence another possible explanation, which adds on the findings by Steeves et al. (2009) discussed above, is the relatively high level of sensation seeking characterizing GL. His sensation seeking score of 27 is more than a standard deviation above the mean in the general population, and very unlike the general PD population, which is less sensation and novelty seeking than healthy controls (Poletti and Bonuccelli, 2012). However, the average sensation seeking score in PD controls resembled that in the general population, though with a large variance. While two PD controls did in fact reveal a low sensation seeking level of 11 and 12, respectively, the remaining two displayed scores very close to the general population of 20 and 23, respectively.

Some limitations must be considered. First, presenting data from a single case does not allow for statistical analyses to be made and general conclusions to be drawn. Nevertheless, the findings are in line with prior findings linking high doses of dopaminergic medication to impaired decision-making and PG in PD as well as implicating the ventral striatum in PG. Secondly, the clinical assessment at follow-up did not include the entire battery of measurements used on day 1, which compromises direct comparisons and limits the conclusions which can be drawn from the study. We were unable to determine whether the level of sensation seeking would have changed with reduction of the medication dose, or whether GL on a lower medication dose would have performed better on the IGT, e.g., by less preference for deck B. Finally, the IGT is not the game of choice for GL and may have resulted in a slightly lower dopamine release following gambling. On the other hand, the expectation of reward is essential in inducing striatal dopamine release in pathological gamblers.

In conclusion, the findings suggest that PG in PD is associated with high doses of a dopamine agonist, long PD duration, pronounced motor symptomatology, a high level of sensation seeking, and risky decision-making associated with an altered dopamine response that reinforces gambling behavior. Thus, the marked decrease in ventral striatal [^11^C]raclopride binding during gambling, observed in GL, implies a gambling-induced dopamine release not seen in PD controls, which may explain how PD patients with PG continue gambling despite personal, social, and financial consequences. Overall, the findings support the concept of dopamine agonist related PG in PD and underline the importance of taking clinical variables, such as age, disease duration, and personality, into account when medicating patients with PD, to reduce the risk of PG.

## Conflict of Interest Statement

The authors declare that the research was conducted in the absence of any commercial or financial relationships that could be construed as a potential conflict of interest.
